# Development of a preliminary *in vitro* drug screening assay based on a newly established culturing system for pre-adult fifth-stage *Onchocerca volvulus* worms

**DOI:** 10.1371/journal.pntd.0007108

**Published:** 2019-01-17

**Authors:** Denis Voronin, Nancy Tricoche, Shabnam Jawahar, Michael Shlossman, Christina A. Bulman, Chelsea Fischer, Michael T. Suderman, Judy A. Sakanari, Sara Lustigman

**Affiliations:** 1 Molecular Parasitology, Lindsley F. Kimball Research Institute, New York Blood Center, New York, New York, United States of America; 2 Dept. of Pharmaceutical Chemistry, University of California San Francisco, San Francisco, California, United States of America; 3 Cell Systems-3D, LLC, Kemah, Texas, United States of America; Imperial College London, Faculty of Medicine, School of Public Health, UNITED KINGDOM

## Abstract

**Background:**

The human filarial parasite *Onchocerca volvulus* is the causative agent of onchocerciasis (river blindness). It causes blindness in 270,000 individuals with an additional 6.5 million suffering from severe skin pathologies. Current international control programs focus on the reduction of microfilaridermia by annually administering ivermectin for more than 20 years with the ultimate goal of blocking of transmission. The adult worms of *O*. *volvulus* can live within nodules for over 15 years and actively release microfilariae for the majority of their lifespan. Therefore, protracted treatment courses of ivermectin are required to block transmission and eventually eliminate the disease. To shorten the time to elimination of this disease, drugs that successfully target macrofilariae (adult parasites) are needed. Unfortunately, there is no small animal model for the infection that could be used for discovery and screening of drugs against adult *O*. *volvulus* parasites. Here, we present an *in vitro* culturing system that supports the growth and development of *O*. *volvulus* young adult worms from the third-stage (L3) infective stage.

**Methodology/Principal findings:**

In this study we optimized the culturing system by testing several monolayer cell lines to support worm growth and development. We have shown that the optimized culturing system allows for the growth of the L3 worms to L5 and that the L5 mature into young adult worms. Moreover, these young *O*. *volvulus* worms were used in preliminary assays to test putative macrofilaricidal drugs and FDA-approved repurposed drugs.

**Conclusion:**

The culture system we have established for *O*. *volvulus* young adult worms offers a promising new platform to advance drug discovery against the human filarial parasite, *O*. *volvulus* and thus supports the continuous pursuit for effective macrofilaricidal drugs. However, this *in vitro* culturing system will have to be further validated for reproducibility before it can be rolled out as a drug screen for decision making in macrofilaricide drug development programs.

## Author summary

The human filarial parasite *Onchocerca volvulus* is the causative agent of onchocerciasis (river blindness). To curb transmission and eventually eliminate the disease, current mass drug administration programs rely on ivermectin that primarily targets the microfilariae, which are released from adult female worms. The female worms, which can live for more than 10–15 years, release millions of microfilariae throughout the course of infection. Thus, to stop transmission of infection and shorten the time to elimination of this disease, a safe and effective drug that kills the adult stage is needed. However, there is no small animal model for an *O*. *volvulus* infection that enables investigators to validate macrofilaricidal drugs. In the present study we describe a newly developed *in vitro* culturing system that supports the growth and development of *O*. *volvulus* young adult worms (OvL5) from the L3 stage and show in preliminary assays that these young worms can be used as an auxiliary for screening putative macrofilaricidal drugs against the ultimate target organism, *O*. *volvulus*.

## Introduction

The human parasitic filarial nematode, *Onchocerca volvulus*, is the causative agent of onchocerciasis (river blindness). Approximately 18 million people are infected with the parasite, mostly in sub-Saharan Africa, and of these individuals an estimated 270,000 people are completely blind, with an additional 6.5 million suffering from severe skin pathologies [1]. Onchocerciasis is a major cause of global disability and is the second leading cause of blindness due to infection, after *Trachoma*. Since 2010, over 3 million disability-adjusted life years have been lost due to *O*. *volvulus* infections [2].

As with all other filarial parasites, *O*. *volvulus* is a vector-borne infection. During a blood meal, the blackfly vector (genus *Simulium*) ingests microfilariae (mf, L1), which develop over 6–10 days and undergo two molts to the infective stage larvae (third-stage, L3). The *O*. *volvulus* L3 (OvL3) migrate to the head and proboscis of the blackfly and are introduced into the bite wound during the blackfly’s subsequent blood meal. These larvae penetrate the wound, migrate deeper into the dermis and subcutaneous tissues of the human host, molt twice from L3 to fourth stage-larvae (L4) and from L4 to pre-adult fifth-stage larvae or L5. Little is known about the time course of this developmental process *in vivo*, but the molt from L3 to L4 occurs in the first 3–7 days [3], while the L4 to L5 molt is estimated to occur after 2 months [4]. Early L5 are considered young adults, and at this stage the worms have partially developed gonads [5]. It takes 279–532 days post infection (dpi) for the closely related *O*. *ochengi* parasite of cattle to develop into fully mature and fertile adult worms capable of releasing mf [6], and more than 400 days post infection (dpi) to do the same in a chimpanzee model for *O*. *volvulus* [4,7]. The majority of the microfilariae migrate to the skin where they are transmitted to the blackfly during a blood meal, thus continuing the life-cycle of the parasite.

Both the adult female (33–50 cm x 130–210 μm) and adult male (19–42 cm x 130–210 μm) worms are encapsulated in collagenous subcutaneous nodules known as onchocercomas [8]. Adult males, however, can migrate within the onchocercomas to fertilize resident females [9]. Fecund females release 1,000–3,000 mf per day, and these first stage larvae called microfilariae (220–360 μm x 5–9 μm) can live for 12–18 months in the human host. Mf are typically found in the skin, and therefore the diagnosis of infection is traditionally made via the microscopic detection of mf released from skin snips, or less commonly by molecular methods such as PCR [10,11].

Current international control programs focus on reducing transmission with the ultimate goal of eliminating onchocerciasis by 2025 in both the Americas and Africa [12]. Currently only ivermectin (IVM) is used for mass drug administration (MDA), but MDA with IVM alone has several limitations. Though IVM does successfully target mf (microfilaricidal), the drug has no effect on adult parasites. Because *O*. *volvulus* can live in the nodules of infected humans for over 15 years and actively release mf for the majority of their lifespan, protracted treatment courses (>20 years) are required to curb transmission and eventually eliminate the disease [13,14]. Additionally, the emergence of IVM-resistant *O*. *volvulus* could greatly hinder this effort [15]. Therefore, despite the challenge of studying a parasite with no small animal model for infection, a critical need still exists for further research to identify novel drug targets, and to develop a new generation of macrofilaricidal drugs against *O*. *volvulus*. One such approach is to target the endosymbiotic bacteria, *Wolbachia*, that reside intracellularly in the parasite. Clinical trials using antibiotic drugs, such as doxycycline or rifampicin, have demonstrated that clearance of *Wolbachia* from the filarial worms results in the slow death of adult worms (macrofilaricidal effect), preceded by sterilization and reduction in transmission frequency [16–19]. Doxycycline treatment is indicated in certain situations, though large scale MDA is limited by both the length of treatment required and contraindications for children and pregnant woman [16,20,21]. Currently, no feasible method exists to permanently sterilize adult female parasites or to kill adult worms outright, highlighting the need for additional research to discover novel macrofilaricidal drug candidates.

In the 1990s, *O*. *volvulus* adult male and female worms, which can only be obtained from infected individuals and after nodulectomy, were used *ex vivo* to screen promising new compounds [9]. Unfortunately, this is not feasible anymore and therefore most of the present screening funnels for novel macrofilaricidal drugs depend on surrogate *in vitro* and *in vivo* filarial models such as *Brugia* spp, *Litomosoides sigmodontis*, and cattle *Onchocerca* spp [21–26]. In the present study we describe a newly developed *in vitro* culturing system that supports the growth and development of *O*. *volvulus* young adult worms (OvL5) from the L3 stage. We show that the optimized culturing system allows the OvL5 to mature into young adult worms that have distinguishable gonads and express adult-specific transcripts. Importantly, we show in preliminary assays that these *in vitro* developed worms can be used as an auxiliary for screening putative macrofilaricidal drugs against the ultimate target organism, *O*. *volvulus*. We also show that the preliminary assays can clearly differentiate inhibitory activities of various FDA repurposed drugs.

## Materials and methods

### Ethics statement

The procedures used for the production of *O*. *volvulus* third-stage-larvae (L3) were approved by an NIH accredited Institutional Review Board of the Medical Research Council Kumba, Cameroon (Protocol 001), and by the Le Comité National d’Ethique de la Recherche pour la Santé Humaine, Yaoundé, Cameroon (Protocol 677). L3 were collected from black flies (*Simulium damnosum*) that were fed on consenting infected donors. The consenting donors were offered and provided with ivermectin treatment by the end of their participation. After seven days the infected flies were dissected and the developed L3 were collected, cleaned and cryopreserved. The cryopreserved L3 were shipped to the New York Blood Center in liquid nitrogen and upon arrival in New York were stored in liquid nitrogen. All protocols using the L3 cryopreserved samples in this study were approved by the New York Blood Center’s IRB (Protocol 321 and Protocol 603–09). All L3 samples were anonymized.

The peripheral blood mononuclear cells (PBMCs) used to culture *O*. *volvulus* L3 were isolated from human leukopaks collected from healthy donors following the New York Blood Center’s approved IRB protocol (Protocol 420). The de-identified human leukopaks were obtained from the New York Blood Center Component Laboratory. The New York City Blood Center obtained written informed consent from all participants involved in the study. All protocols were conducted in accordance with National Institutes of Health guidelines for the care and use of human subjects.

### Culturing of *Onchocerca volvulus in vitro*

Cryopreserved *O*. *volvulus* L3 stage larvae (OvL3) were thawed and washed in “larvae” medium that contains 1:1 NCTC-109 and IMDM supplemented with Glutamax (1x) and 2x Antibiotic-Antimycotic (Life Technologies). After washing, the larvae were dispersed in a 96-well plate (10 larvae/well) containing 1.5x10^5^ human Peripheral Mononuclear Blood Cells (PBMC) per well in complete “larvae” medium, which was supplemented with heat inactivated (HI) 20% fetal bovine serum (FBS, Sigma). Worms were cultured at 37 °C with 5% CO_2_ until day 6 when the molting rate was estimated under an inverted microscope based on the presence of the highly motile L4 (OvL4) and the empty L3 cuticle.

**Fig 1:**
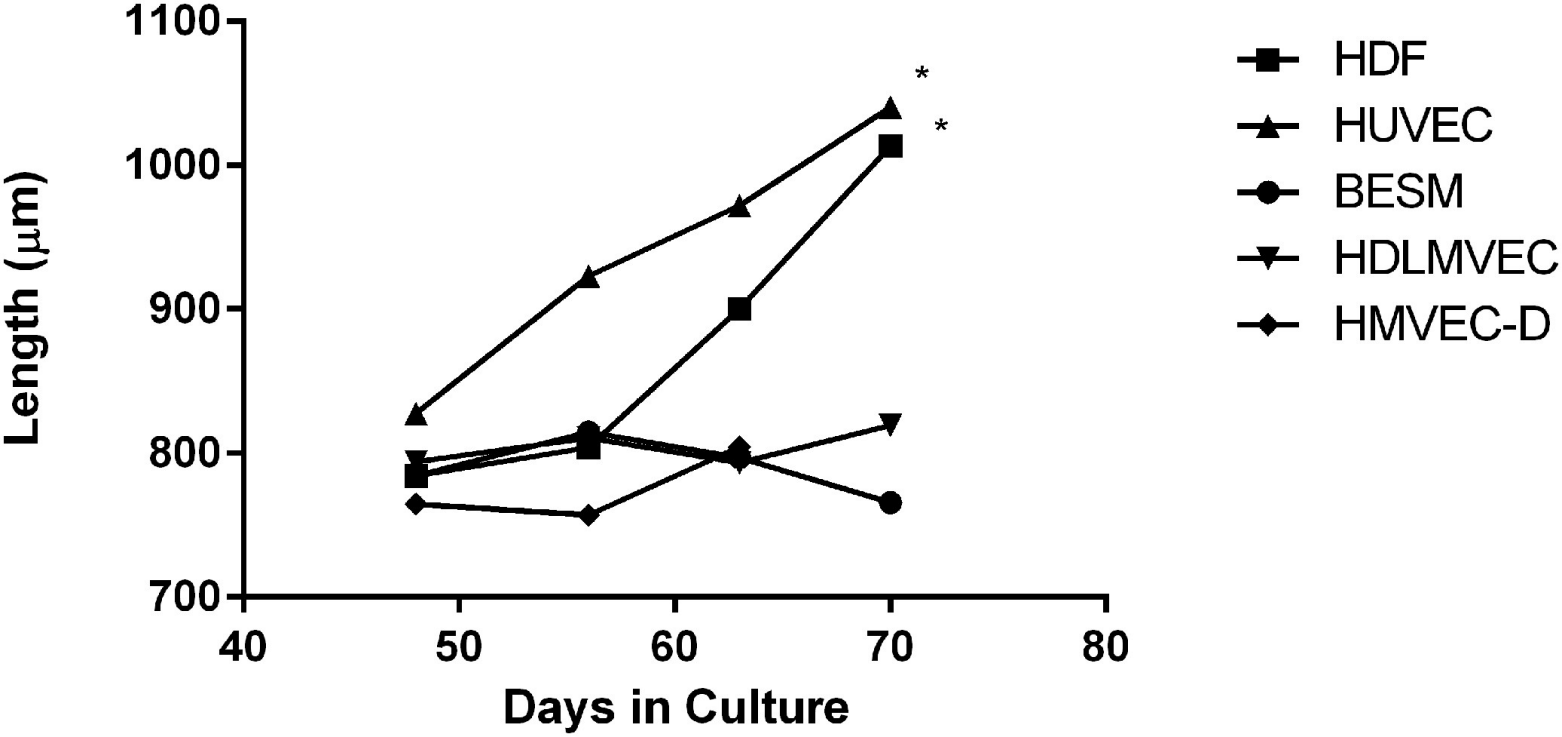
Dynamic OvL4-L5 growth in 96-well plate settings. HDF and HUVEC showed the best support for worms’ growth (day 70: grown on the HDF monolayers—range of 803–1374 μm, *p* = 0.03 (n = 7) as compared to BESM; grown on HUVEC monolayers—range of 804–1504 μm, *p* = 0.03 (n = 7) as compared to BESM). L4 cultured in HMVEC-D, BESM or HDLMVEC had the lowest growth rate (ANOVA test *p* = 0.016 (n = 9), d56) and highest mortality. *—*p*<0.05.

To culture OvL4 long-term, we first tested several monolayer cell lines: Human Umbilical Vein Endothelial Cells (HUVEC), Human Dermal Fibroblasts (HDF), Human Skeletal Muscle Cells (HSkMC), Human Dermal Microvascular Endothelial Cells (HMVEC-D), Human Dermal Lymphatic Microvascular Endothelial Cells (HDLMVEC), and Keratinocytes, all purchased from Lonza Inc. (Allendale, NJ). Each of the cell lines were maintained in the laboratory in their respective recommended base media. The motile OvL4 larvae were transferred into new 96-well plates that had been seeded with the various cell lines. The culturing medium consisted of 50:50 “larvae” media to cell media, supplemented with 20% HI FBS and 1x Antibiotic-Antimycotic. The growth of the larvae was measured over 70 days; the two cell lines that supported the best growth of the OvL5 were HUVEC and HDF (Fig 1). Significance was determined using ANOVA (day 56) and a t-test (day 70).

To prevent OvL4 from becoming entangled within the cell monolayer in the 96-well plates, we devised an alternative culturing system where ~10 OvL4 were placed inside a transwell (Corning Transwell, 6.5 mm Transwell with 3.0 μm pore polyester membrane insert, Sigma CLS3472) in 24-well plates seeded with HDF or HUVEC monolayers at 1x10^4^ cells/well in 1 ml of OvL4-media. This media was comprised of 1:1 NCTC-109: MEM-alpha with either DMEM/F12 (Life Technologies) or EBM-2 (Clonetics EBM-2 Lonza CC-3156), the recommended base media for each cell line at 60:40 ratios. The OvL4-media was further supplemented with 20% HI FBS, 1x Antibiotic-Antimycotic, 1% Glucose (Sigma G8769) and 1% Sodium Pyruvate and 1% ITS (Life Technologies 11360–070 and 51500–056). Unfortunately, some of the HDF cells present in suspension tended to migrate over the outer membrane of the transwell during the media change and to overgrow with time, causing the worms to become entangled. Therefore, to maintain worms free of any cell entanglements, the subsequent and final cultures, including the screening assays, were set up using only the HUVEC monolayer. To further optimize culturing conditions, we first tested the outcomes of culturing the worms after adding 0.1% Lipid Mixture-1 (Sigma L0288) to the OvL4-media. This medium was named complete OvL4 (OvL4-CM) medium. OvL4s were maintained for at least 104–120 days within the transwells with 3 media exchanges per week: 0.5 ml of old medium was removed, and 0.5 ml of fresh media was added. Fresh HUVEC monolayers were prepared weekly and the transwells containing the developing OvL4 were transferred once a week into freshly seeded monolayers with 1 ml of OvL4-CM.

To further augment the growth of the developing worms, the OvL4-CM medium was also supplemented with 1% Non-Essential Amino Acids (Life Technologies 11140–050), and 25% HI FBS (instead of 20%). This newly devised media composition (OvL4-CMS) produced worms with the longest length thus far, with the longest worm measuring > 3,000 μm (See Results). Significance was determined using a t-test.

### Microscopy

The measurements, images, and videos of live larvae were taken on a Nikon Eclipse TS100 inverted microscope equipped with a Nikon DS-Fi2 camera controlled by the NIS Elements version 4.3 Windows based imaging program.

For transmission electron microscopic analyses of the developing worms, samples of OvL4 (n = 10) on days 48–50, molting OvL4 (n = 10) on days 50–60, OvL5 (n = 10) on days 60–75 or 120 were fixed with 2.5% glutaraldehyde and 2% paraformaldehyde in sodium cacodylate buffer (0.1M) for 2 h. After fixation, worms were washed three times in sodium cacodylate buffer (0.1M) and post-fixed with 1.5% OsO_4_ for 1 h. Worms were then washed and dehydrated using a series of increasing ethanol concentrations (50–100%) with a final wash with propylene oxide. Samples were then embedded in plastic resin (Epon 812, EMS USA) and prepared for sectioning. Ultrathin sections were contrasted with UranyLess (EMS, USA) and lead citrate and then were analyzed under the Tecnai G2 Spirit transmission electron microscope.

### RNA isolation, cDNA synthesis and PCR

Ten (n = 10) developing OvL4 D21, 10 OvL5 D76 and 10 OvL5 D96 were collected from *in vitro* cultures. Worms were washed with PBS and stored at -80°C. OvL4 and OvL5 were collected four times for separate RNA extractions and analysis. Two aliquots, 50 OvL3 each, were prepared from thawed and washed cryopreserved larvae collected from black flies. One pool of adult RNA was prepared from 1 female and 10 male worms that were frozen. Total RNA was extracted from the collected worms by a TRIzol-based method followed by further purification using a column based mini RNA extraction kit (Invitrogen). During purification RNA was treated with DNase I (Invitrogen) on columns according to the manufacturer’s instructions. Purified RNA was used as a template for cDNA synthesis performed using the SuperScript III first-strand synthesis system (Invitrogen). cDNA synthesized from adult worms was diluted 100 times with water. The cDNA was amplified by PCR using primers for *O*. *volvulus* transcripts corresponding to OVOC11951, OVOC5433, OVOC9683, OVOC12838, OVOC2456 (S1 Table), which were selected based on their overexpression in *O*. *volvulus* adult worms vs. mf and OvL3 [27]. For the positive control we used primers for Ov-Tubulin. Primers for an intron were used as a negative control to test that there was no contamination of genomic DNA. PCR products were run using 1.5% agarose gel electrophoresis.

### Drug screening of OvL5 worms *in vitro*

A week prior to performing an assay, cultured OvL5s were transferred from the transwells into a small petri dish containing fresh OvL4-CMS. Aliquots of 8–10 worms were retrieved from the petri dish and placed inside new transwells over newly seeded HUVEC monolayers in OvL4-CMS. The HUVEC cell monolayers (1x 10^4^/well of a 24-well plate, with 0.5 ml of OvL4-CMS) were prepared as described above one to two days prior to the drug screening assay. After transferring OvL5 to these plates, test drugs (flubendazole or oxfendazole) were added to each well as needed. Tested compounds were dissolved in DMSO at a concentration of 10 mM and added to the OvL5 wells in 0.5 ml OvL4-CMS containing 2x the desired final concentration. Plates were incubated at 37°C in a 5% CO_2_ incubator. Media containing freshly prepared compound was replaced every 2–3 days for the 14 days of treatment (or less depending on the assay) by removing 0.5 ml of the media and adding 0.5 ml of the appropriate treatment media to maintain the concentration of a drug. For the normal growth control, we used complete media with a final concentration of 0.05% DMSO. After the treatment period (14 days or less), the media was replaced completely with 1 ml of OvL4-CMS, and then every 2–3 days with fresh media as described above for 14 additional days. Motility was recorded every 2–3 days over the full period of the assay according to the following scale: 100% motility, constant coiling movement; 75% motility, slower coiling; 50% motility, slow and intermittent movement; 25% motility, very slow movement or twitching; and 0% motility, no movement (S1 Video). Observations were done blinded by two individuals. On Day 28, viability was assessed by MTT staining as previously described [28]. Untreated control and treated OvL5 within the transwells were washed with PBS and then incubated with MTT (3-(4,5-Dimethylthiazol-2-yl)-2,5-Diphenyltetrazolium Bromide) (0.1%) in PBS at 37°C under 5% CO_2_ for 1 hour, followed by an additional wash of the parasites with PBS. The product of MTT reduction (formazan) is blue. Worms were considered dead if no staining or < 50% within the worm was observed using an inverted microscope (S1 Fig). Worms stained blue or > 50% stained were considered alive. All test treatments were performed in duplicates. Significance was determined using a t-test.

To determine the IC_50_ for drug activity on OvL5, serial 2X concentrations of the test compounds (60, 20, 6, 2, 0.6, and 0.2 μM) were prepared in OvL4-CMS medium. The 0.5 ml of media containing the 2x compound was added to transwells containing OvL5 worms (n = 10, 75–80 days or less based on the assay) in 0.5 ml of media to yield final test concentrations of 30, 10, 3, 1, 0.3, and 0.1 μM. The doses of flubendazole used were in the same range as previously done with adult *B*. *malayi* worms *in vitro* [29]. Moreover, in previous *in vivo* studies of flubendazole in rats, doses of 65–402 mg/kg that corresponded to C_max_ = 0.90–1.33 μg/ml were used [30]. In our studies we used flubendazole at the concentration used *in vivo* (2 μM), which is equivalent to 0.62 μg/ml. OvL5 (n = 20) cultured in the presence of a final concentration of 0.05% DMSO in media served as a control for normal development. All test treatments were performed in duplicate. Plates were incubated at 37 °C in a 5% CO_2_ incubator. Media replacement with or without compounds followed the same procedure described above. Motility was monitored over the full course of treatment and thereafter, with IC_50_ for motility determined on the last day of culturing. Viability was determined the following day. The percent inhibition of motility and viability for treated OvL5 worms were calculated with respect to the movement and viability of OvL5 in wells containing the DMSO in normal complete media. IC_50_ values were calculated using Graph Pad Prism v6.0 (http://www.graphpad.com). The probability of assumption (R^2^) was calculated by the software and is reported in each graph.

### Drug screening of OvL3 for molting inhibition *in vitro*

Efficacy of drugs on *O*. *volvulus* L3 larvae using the molting assay was performed as described previously [31]. Briefly, 5–10 L3 per 50 μL in complete medium (CM) containing 20% heat inactivated FBS were distributed to 10 wells of a 96-well plate and 1.5 × 10^5^ normal PBMCs were added per well in 50 μL. 2X dilutions of a compound (final concentration of 30, 10, 3, 1, 0.3, 0.1 and 0.03 μM) were then added to each well, 100 μL per well. The total number of worms tested for each treatment condition was 30 and 60 for controls. Controls of DMSO in complete medium and complete medium without DMSO were included in each assay. The concentration of DMSO within the cultures was never higher than 0.05%. The 96-well plates were then incubated at 37 °C in a 5% CO_2_ incubator until day six when molting was recorded using an inverted microscope and observed for the presence of the fourth-stage larvae (L4) and the empty casts of the L3. The probability of assumption (R^2^) was calculated by the software and is reported in each graph.

### Drug screening of adult *Brugia* worms *in vitro*

Adult female *B*. *pahangi* were assayed using methods described by Marcellino *et al*. (2012) and Bulman *et al*. (2015) [23,26]. Individual females were placed in each well of a 24-well plate with complete media Extra media was removed from plates using a Biomek FxP, leaving 500 μL of media per well. Auranofin, niclosamide, and nitazoxanide were dissolved in DMSO (Sigma) and 10 mM stock solutions were stored at -20°C. Drugs were tested using 4 worms per compound per concentration. Plates were kept in a 37°C, 5% CO_2_ incubator for 3 days. 6-point serial dilutions were used to determine IC_50_s: for auranofin the concentrations were 10 μM, 3 μM, 1 μM, 0.3 μM, 0.1 μM and 0.03 μM; for niclosamide and nitazoxanide the concentrations were from 30 μM to 0.1 μM; 1% DMSO was used as a control. These concentrations were selected based on published data [26]. Non-linear regression curves were used to calculate IC_50_ values using Graph Pad Prism v6.0. IC_50_ assays were repeated 2–3 times and the means with R^2^ values greater than or equal to 0.7 are reported.

## Results

### The development of *O*. *volvulus* L4 to L5 *in vitro*

The preparation of OvL4 followed our standard protocol using cryopreserved L3 cultured in complete ‘larvae’ medium in the presence of human PBMCs [26,32]. As expected, *O*. *volvulus* L3 molted to L4 within the first 6 days of culture; molting of L3 was confirmed by detecting empty L3 casts within the well and observing highly motile OvL4 (S2 Fig). In general, 30–60% of the OvL3 molted to OvL4. The varied molting rate was associated with the numerous lots of the cryopreserved, thawed *O*. *volvulus* L3. The L3 stage larvae had a median length of 647 μm (ranged from 558 μm– 708 μm), while the newly molted L4 had a median length of 703 μm (583 μm– 810 μm). In our initial attempts to keep OvL4 for long term culturing, we kept the newly molted OvL4 in the presence of freshly cultured PBMCs but this proved unsuccessful, as larvae did not thrive more than a week later in the culture.

**Fig 2:**
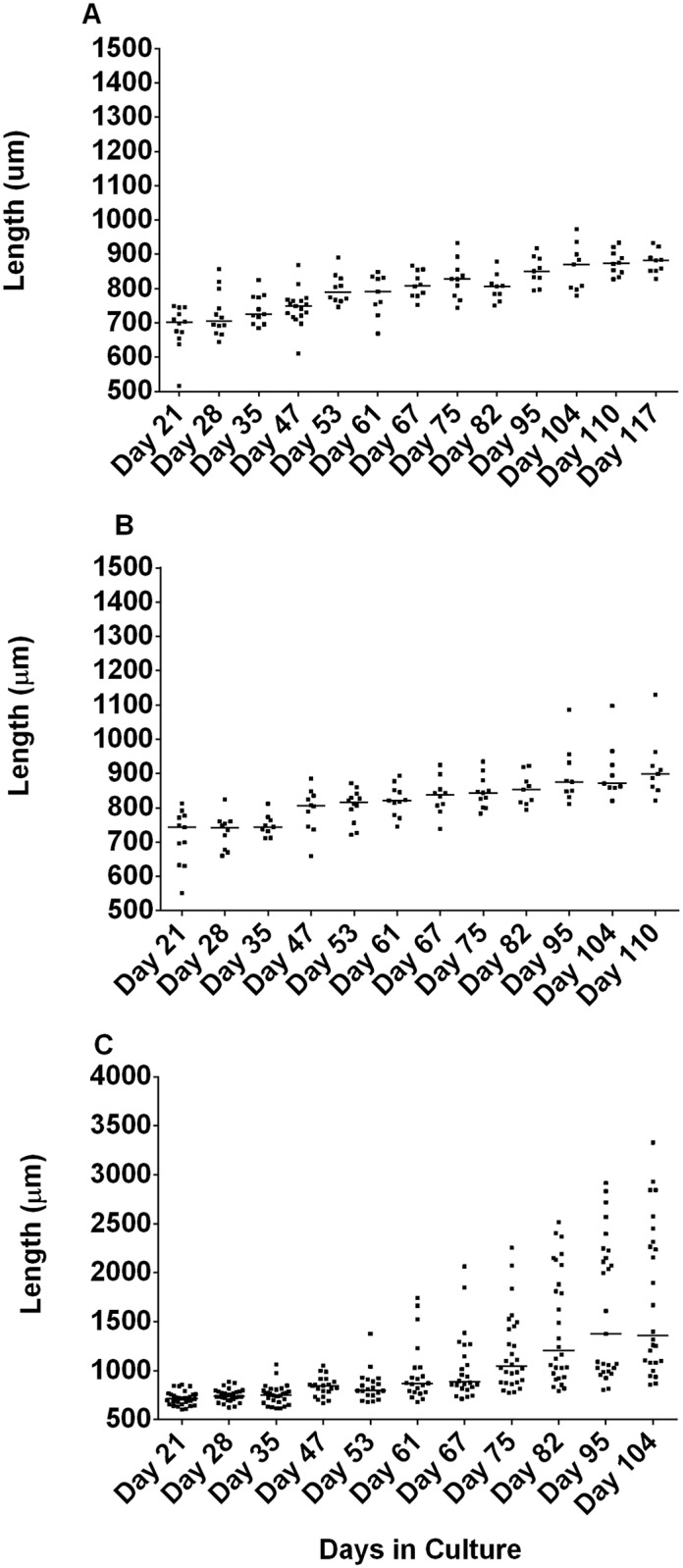
Comparing growth of OvL4 to OvL5 in 24-well plate containing a monolayer of HUVEC and in the presence of varying culture media composition. A. OvL4-media with 20% FBS; B. OvL4-media with 20% FBS and 0.1% lipid mixture (OvL4-CM); C. OvL4-media with 25% FBS, 0.1% lipid mixture, and 1% mix of amino acids (OvL4-CMS). OvL5 cultured in OvL4-CMS media had the longest length (on day 104 –range of 857–3329 μm, median 1358 μm; *p* = 0.002 (n = 26) as compared to the length of worms in OvL4-CM media with 20% FBS (A), *p* = 0.003 (n = 26) as compared to the length of worms in OvL4-CM media with 20% FBS and 0.1% lipid mixture (B)).

We then decided to investigate which human cell lines would best support OvL4 growth. We cultured L4 (n = 10 per cell line) in 96-well plates seeded with six different cell line monolayers; HMVEC-D, HUVEC, HDF, HSkMC, BESM, and LMVE. L4 cultured in HMVEC-D, BESM or HDLMVEC had the lowest growth (p = 0.016 (n = 9), d56) and highest mortality rate (Fig 1). In comparison, the HUVEC and HDF monolayers showed promise, with growth continuing after day 56 (Fig 1). To further optimize media composition all experiments were performed with the HUVEC monolayers only using 24-well plates containing transwells as described above (Fig 2). This was based on two observations. The median length of OvL5s maintained on HUVEC monolayers was slightly longer on day 70 (range of 804–1504 μm, median 1040 μm), even if not statistically longer than those grown on the HDF monolayers (range of 803–1374 μm, median 1013 μm) (Fig 1). Additionally, it prevented the logistical challenges with HDF cells, which overgrew within the cultures, causing the growing OvL5 to become entangled within the cellular monolayer, and subsequently negatively affecting the fitness of developing worms. This culturing system, the HUVEC monolayers in a 24-well plate containing the larvae in the transwells, was used to further refine our media composition and establish whether supplements c improve the growth of OvL5 (Fig 2A). The first supplement tested was the addition of 0.1% lipid mixture (Fig 2B). This was followed by the addition of 1% Non-Essential amino acids to the 0.1% lipid mixture and an increase in the final concentration of FBS from 20% to 25% (Fig 2C); this modified media is referred as OvL4-CMS.

**Fig 3:**
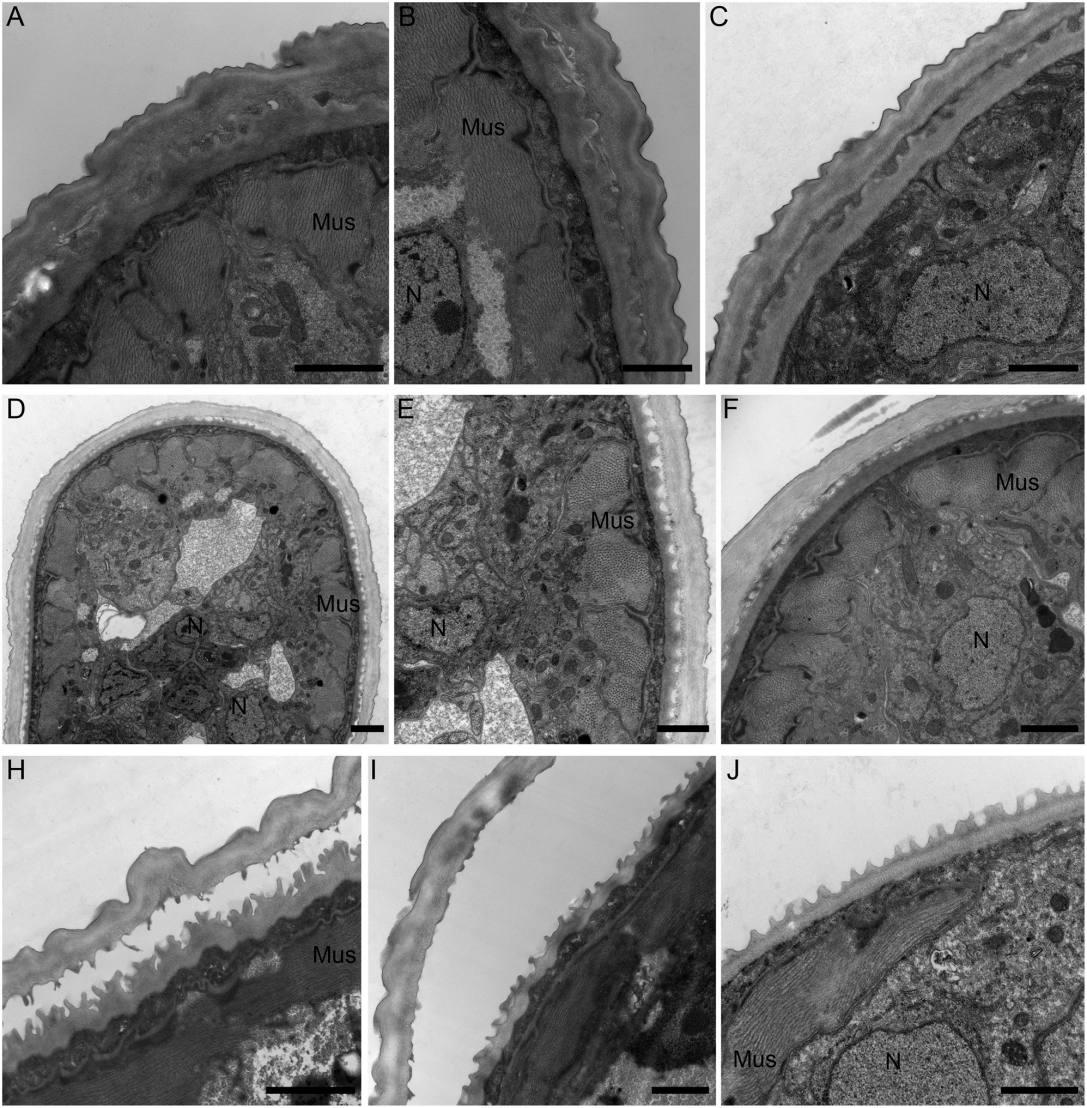
Transmission electron microscopy of OvL4 during molting to OvL5. A-C, Growth of new cuticle (c) beneath the old one (arrowheads) (day 48–50); D-F, beginning of cuticle separation (worms on day 50–60); H-J, final stage of cuticle separation (worms on days 60–75). Worms also had well-developed muscle filaments (Mus), and normal morphology of cells. Abbreviation: N—nuclei. Bar: A-C– 1 μm; D– 2 μm; E-J– 1 μm.

As observed before in 96-well cultures (Fig 1), the OvL4 in OvL4-CMS (Fig 2C) as well as those in OvL4-media and OvL4-CM media (Fig 2A and 2B) grew slowly until day 53 (range 678–1376 μm, median 800 μm) after which time, there was an increase in worm length between day 61 (680–1743 μm, median 871 μm) and day 75 (range of 776–2254 μm, median 1046 μm) that coincided with molting of L4 to L5 (Fig 3C). By day 104 the worms that were cultured in OvL4-CMS were much longer (range of 857–3329 μm, median 1358 μm) than in the other two media systems (p<0.01 (n = 26)). As seen clearly in Fig 2C, two clusters of worms were observed, those larger than 1358 μm and those that were smaller than 1358 μm (median). Sexual dimorphism could explain this growth difference. Notably, starting on day 75 we found that it easier to distinguish between male and female worms in the same culture well (male worms were generally smaller than female worms). The sharp increase in growth on day 75 (Fig 2C) and thereafter might be also attributed to completion of the molting process from OvL4 to OvL5 as evidenced by structural analysis using transmission electron microscopy (Fig 3).

**Fig 4:**
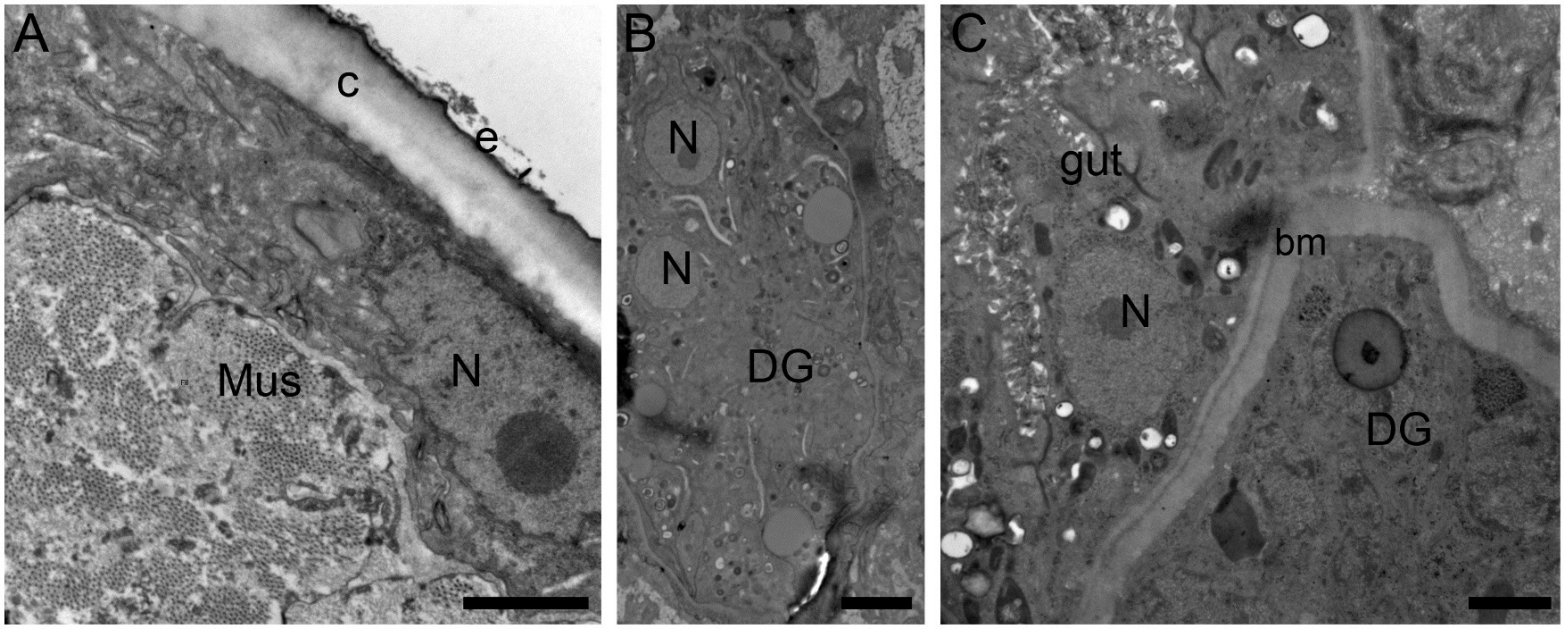
Microphotographs of *O*. *volvulus* (120 days in culture) show normal morphology of cells and developing organs. A, worms have a cuticle and epicuticle layer, also showed normal organization of muscle filaments (Mus). B, C, young adults have germ cells organized in developing gonads (DG) with basal membrane (bm). Abbreviation: N—nuclei. Bar A, C– 1 μm; B– 2 μm.

The L4 to L5 molting process is clearly evidenced by the presence of a separation between the cuticles of L4 and the developing L5 worms on days 48–60; the molting process was completed by days 60–75 (Fig 3). Notably, the developing L5 had a highly annulated cuticle underneath the L4 cuticle (Fig 3H–3J), and the OvL5 (day 120) had a distinguishable epicuticle, gut, and developing gonads (Fig 4).

### Molecular markers verify that the OvL5s are pre-adult worms

**Fig 5:**
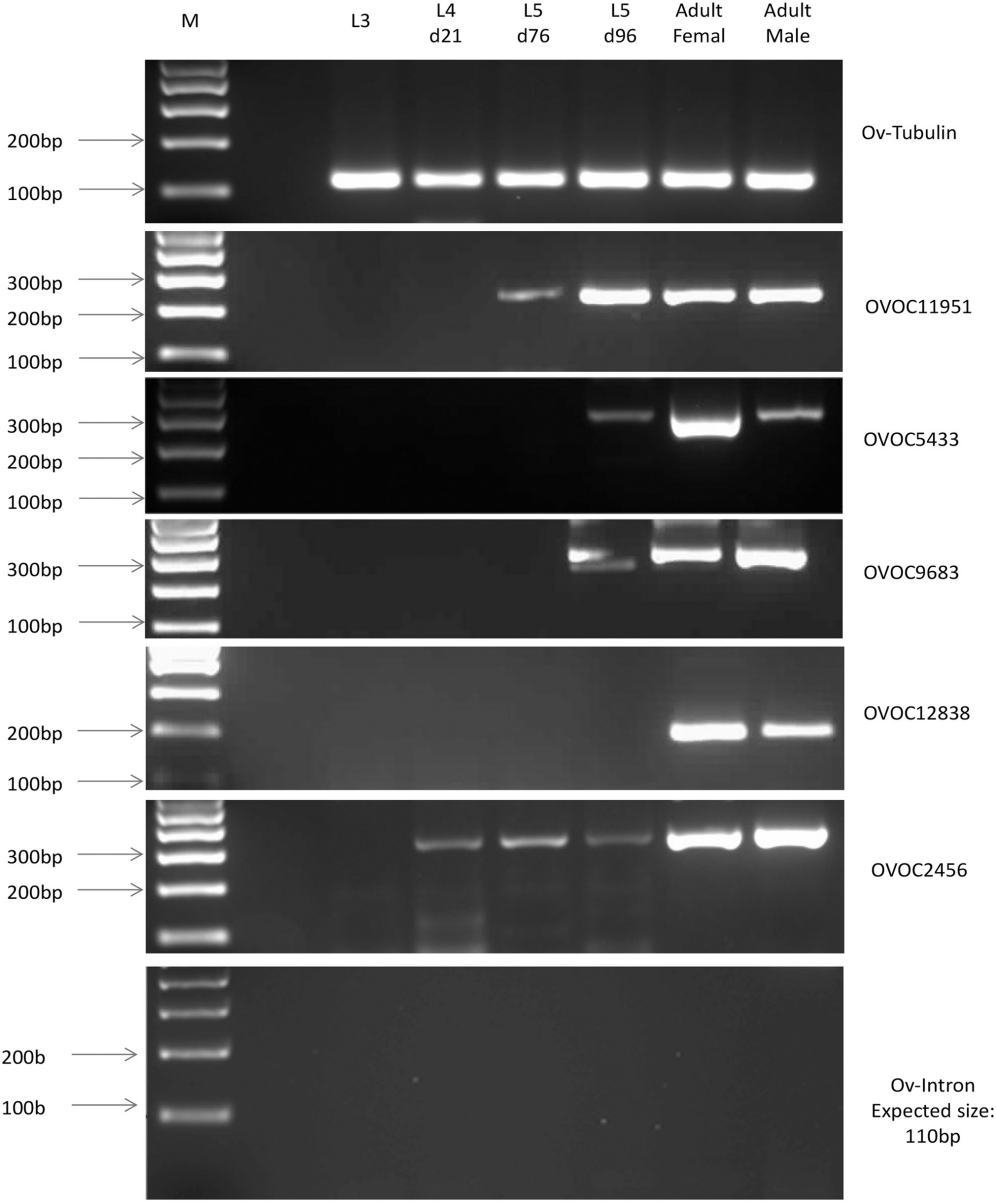
Gel electrophoresis of PCR products obtained using cDNA prepared from L3, L4 D21, L5 D76, L5 D96, adult males and females, and primers corresponding to selected stage-specific biomarkers (S1 Table). Tubulin was used as quality control for cDNA and PCR. Primers for an intron (expected product size– 110 bp) were used as a negative control to test that there was no contamination of genomic DNA. M—Marker.

The stage-specific transcriptomic and proteomic datasets for *O*. *volvulus* L3, L4, and adult males and females [27] were analyzed to select transcripts that appear specifically and/or exclusively expressed in adult worms. Five genes expressed predominately in adult worms were selected for analysis of their expression in OvL3 collected from blackflies (D0), OvL4 (D21), OvL5 after molting (D76), and pre-adult OvL5 (D96) in comparison to their expression in female and male adult worms recovered from nodules collected from humans after nodulectomy. The expression of tubulin in each stage was used as a positive control. As seen in Fig 5, the transcript of OVOC11951 was specifically expressed on day 76 and day 96 OvL5 and in adult worms, while the OVOC5433 and OVOC9683 genes were expressed only in the pre-adult OvL5s (D96) and in adult female and male worms. The OVOC2456 gene, by comparison, was already expressed in L4 day 21 and its expression was continuously present in the developing L5 and in adult worms. The OVOC12838 gene was, however, expressed only in the adult female and male worms. None of the transcripts, except tubulin (control of the quality of cDNA), were expressed in OvL3 as predicted by the transcriptomic dataset.

### The use of the OvL5 culturing system for drug screening

**Fig 6:**
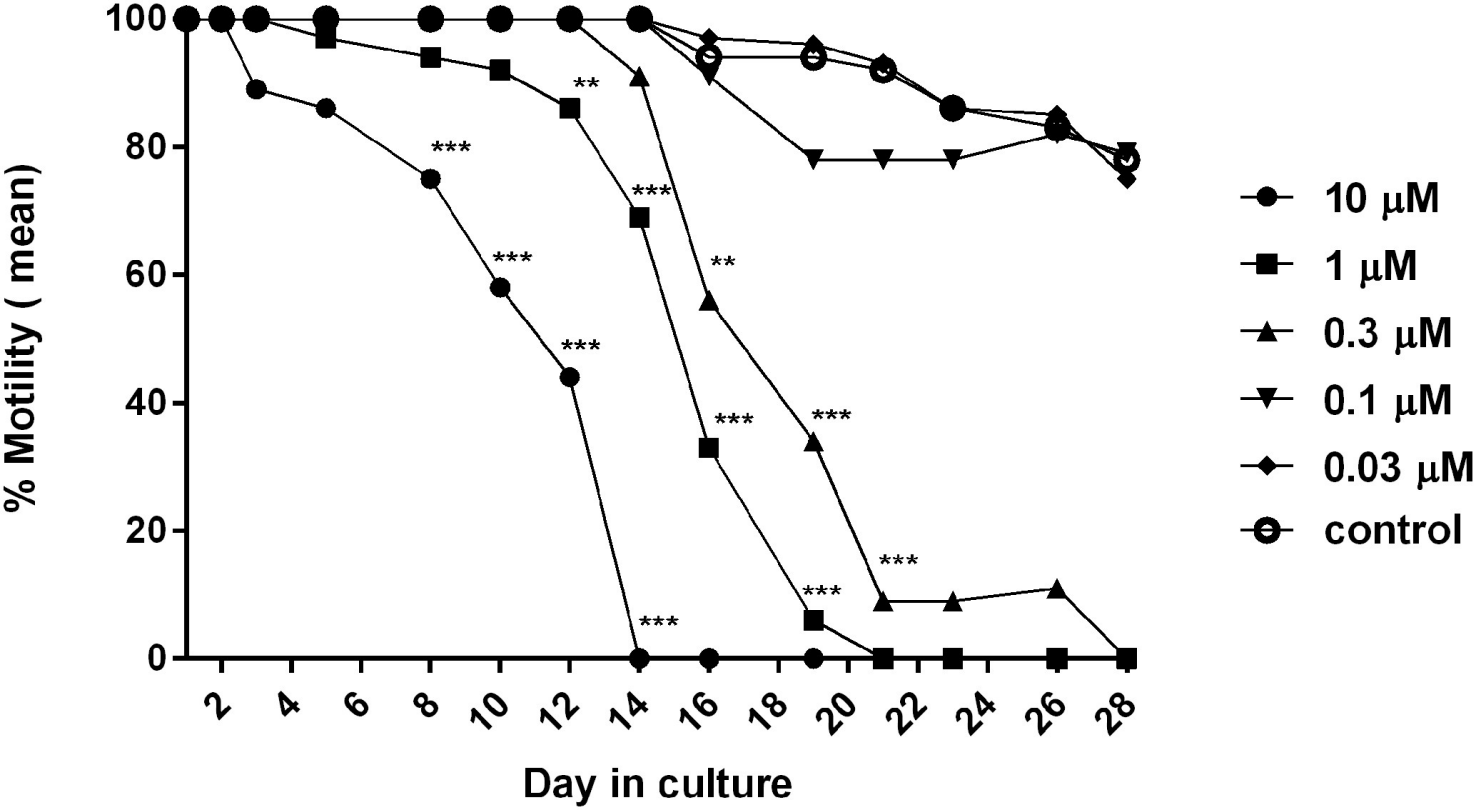
Motility of *O*. *volvulus* L5 worms exposed to different concentrations of flubendazole. Treatment was for 14 days with an additional 14 day follow up without the drug. Flubendazole (0.3 μM and higher) caused significant inhibition of worms’ motility. Specifically: 10 μM of the drug caused significant (***—*p*<0.001, n = 9) inhibition of motility as compared with control (n = 14) starting on the 9^th^ day; 1 μM of the drug caused significant (**—*p*<0.01, n = 11) inhibition of motility as compared with control (n = 14) starting on the 12^th^ day; and 0.3 μM of drug caused significant (*p*<0.01, n = 8) inhibition of motility as compared with control (n = 14) starting on the 16^th^ day. Motility was observed using a microscope and scored based on the following scale: 100% motility, constant coiling movement; 75% motility, slower coiling; 50% motility, slow and intermittent movement; 25% motility, very slow movement or twitching; and 0% motility, no movement.

Once our *in vitro* culturing system for OvL5 was optimized, we tested whether this newly developed system could be applied to macrofilaricidal drug screening assays. We first tested flubendazole, a known macrofilaricidal drug for *O*. *volvulus* infection [33,34]. Since we were able to maintain OvL5 for up to 104 days in culture, we decided to test the OvL5 worms (day 73) with various concentrations of flubendazole (0.03–10 μM) until we first observed effects on motility. The drug was then removed (media replacement) and observations of worm motility continued over an additional period of culturing. By day 14, 1 μM flubendazole started to have a noticeable effect on motility (~40% inhibition, p<0.01, n = 11). We therefore decided to stop the treatment on day 14 for all treatment wells and followed worm motility in the presence of normal media for an additional 14 days (Fig 6). The IC_50_ for motility was determined on day 28 and for viability (MTT assay) on day 29 (S3 Fig). The IC_50_ was 0.13 μM for both the inhibition of motility and for the inhibition of viability (S3A and S3B Fig). When oxfendazole, another benzimidazole drug previously shown to have an *in vivo* macrofilaricidal activity against several other filarial species in animal models [35], was tested on OvL5 (day 80), we found some differences based on the phenotype analyzed. The IC_50_ for inhibition of motility was similar to that of flubendazole (0.12 μM, (S3C Fig)), however, the IC_50_ for inhibition of worm viability was 0.54 μM (S3E Fig)—4 times higher than after the treatment with flubendazole.

Second, we tested the effects of three FDA repurposed drugs (auranofin, niclosamide and nitazoxanide) on the molting of OvL4 (D57) and their development into OvL5 (D63); OvL4 on D57 in culture were used in this drug screening experiment. Auranofin, a gold-containing drug used for rheumatoid arthritis, has been shown *in vitro* to inhibit the molting of L3 of *O*. *volvulus* and inhibit the motility of adult female *Brugia pahangi*, with IC_50_ values in the low μM range of 0.3 and 0.5, respectively [26]. Niclosamide is an orally bioavailable chlorinated salicylanilide used as an anthelmintic drug against tapeworm infection. In our *in vitro* screens with *O*. *volvulus* L3 and *B*. *pahangi* female worms, niclosamide inhibited molting of *O*. *volvulus* L3 with an IC_50_ of 0.08 μM (S4 Fig), while it inhibited the motility of the *B*. *pahangi* female adult worms with an IC_50_ of 1.2 μM (Table 1). Nitazoxanide, a broad-spectrum antiparasitic drug, was less effective on *O*. *volvulus* L3 (S4 Fig) and *B*. *pahangi* female adult worms; IC_50_ of 4.71 and 5.8 μM, respectively (Table 1).

**Table 1:** The effects of auranofin, niclosamide and nitazoxanide (IC_50_) on filarial worms cultured *in vitro*.

Drug	Inhibition of *O*. *volvulus* L3 molting (μM), n = 30	Inhibition of motility of *B*. *pahangi* female adult worms (μM), n = 4	Killing of *O*. *volvulus* L5 (μM), n = 30
**Auranofin**	**0.3 (r**^**2**^ **= 1.0)**	**0.5 (r**^**2**^ **= 0.961)**	**1.0 (r**^**2**^ **= 0.965)**
**Niclosamide**	**0.08 (r**^**2**^ **= 0.952)**	**1.2 (r**^**2**^ **= 0.933)**	**1.5 (r**^**2**^ **= 0.879)**
**Nitazoxanide**	**4.7 (r**^**2**^ **= 0.833)**	**5.8 (r**^**2**^ **= 0.917)**	**24.0 (r**^**2**^ **= 0.977)**

Using our *in vitro* OvL5 assay, two of these three drugs (auranofin, niclosamide) were shown to be effective in killing the pre-adult worms after a 6-day treatment *in vitro*: 3 μM auranofin killed 100% of the *O*. *volvulus* worms and 3 μM niclosamide killed 88% of the *O*. *volvulus* worms. In comparison, 30 μM nitazoxanide killed only 65% of the *O*. *volvulus* worms. The corresponding IC_50_ values for viability were 1.0 μM for auranofin, 1.5 μM for niclosamide, and 24.0 μM for nitazoxanide. The range of IC_50_ values for the OvL5 appear to be somewhat similar to the μM range observed using adult *Brugia* assays, thus supporting the validity of this *in vitro* model for future screening of novel macrofilaricidal drugs against *O*. *volvulus*.

## Discussion

We have developed, optimized, and tested a novel *in vitro* culturing system that supports the growth and development of *O*. *volvulus* young adult worms from the L3 stage. It shows that the OvL4 worms can be optimally maintained in OvL4-CMS medium containing supplements (a mix of amino acids, lipids and 25% FBS) with a supportive monolayer of HUVEC (Fig 2C). Using this culturing system, not only the molting of OvL4 to L5 is supported, but also the subsequent morphogenesis into young pre-adult stages. Filarial nematodes molt their cuticles (an extracellular hydroskeleton that overlays the hypodermis of worms) four times during pre-adult development. Here, we observed that worms developing from OvL4 to OvL5 in the *in vitro* setting shed their old cuticle by first building a new one beneath (Fig 4). Development of *O*. *volvulus* beyond the fourth-stage larval stage has previously been attempted in a few animal models, including mice, non-human primates, and chimpanzees, using the implantation of diffusion chambers containing the larvae. The greatest increase in the length of the recovered worms was after 4 weeks—from 350 μm to 424–601 μm in DBA/2J mice and up to 559–759 μm in chimpanzees [5]. These *in vivo* models did not support further worm development past 63 days [5]. In our optimized *in vitro* culturing system, we were able to culture worms for more than 120 days and the worms showed continuous growth up to 3329 μm (range of 857–3329 μm, day 104) (Fig 2C).

According to a recently published datasets of the transcriptome of *O*. *volvulus* and the transcript levels in the worms during different stages of development, a few candidate transcripts were identified that could be used as potential biomarkers of adult stages of development [27]. We assumed that these biomarkers could be also employed to monitor the progress of worm development in our *in vitro* system. We found that three (OVOC11951, OVOC5433, and OVOC9683) of the five selected adult–stage-specific transcripts were also expressed by young adults developed in our culturing system (Fig 5). These three transcripts could be therefore used as potential markers to monitor the efficacy of treatments performed using the *in vitro* system for *O*. *volvulus* drug screening. Moreover, TEM analysis of OvL5 (D120) showed the presence of an epicuticle that appears to be characteristic of adult worms (Fig 5), this further confirming that the OvL5s developed *in vitro* are pre-adult worms.

The most important application of our ability to culture OvL4 and OvL5 *in vitro* is the promising novel capacity to screen compounds (including repurposed drugs) against the target filarial parasite *O*. *volvulus*. In preliminary assays we tested the effects of few putative macrofilaricidal drugs on the motility and viability of the *in vitro* developed young *O*. *volvulus* worms or on the OvL4-OvL5 molting process. Both effects (motility/ viability of OvL5 and molting to OvL5) are important, as an ideal drug candidate would arrest the development of growing worms in addition to their macrofilaricidal activity on adult worms within the nodule. By employing our novel long term *in vitro* culturing system we were able to estimate initial IC_50_ values for several repurposed drugs including flubendazole, a known macrofilaricidal drug candidate for *O*. *volvulus* infection [33,34]. In comparison, when other *in vitro* screening models were used such as that with *B*. *malayi* adult worms, flubendazole did not inhibit worm motility after 5 days, although it caused some damage [29]. With the other *in vitro* screening models, worm motility is limited to only a few days (7 days at the most using the adult female *O*. *ochengi* worms). *In vivo* it takes 42 days until the effects of flubendazole can be observed on implanted *B*. *malayi* adult worms [36]. Because we were able to optimize the long-term culturing of OvL4 and OvL5, we were able to discover that only after the OvL5 were treated with flubendazole for 14 days a significant reduction in their motility (0.3 μM and higher) could be observed. As our *in vitro* model contains also human cells (feeder layer), it is possible that flubendazole can be metabolized to hydrolyzed flubendazole [30,37]. It is promising that a flubendazole metabolite might also has an intrinsic activity against filariae [34]. The initial IC_50_ value (S3 Fig) of oxfendazole, a sulfoxide metabolite of fenbendazole, and a macrofilaricidal drug used against several filarial species in animal models [35], was also obtained for OvL5 and is similar to that of flubendazole.

Additionally, this *in vitro* culturing system allowed us to analyze the activity of a set of other FDA repurposed drugs (auranofin, niclosamide and nitazoxanide) against *O*. *volvulus*. It had been previously shown that auranofin was effective at killing adult *Brugia* and *O*. *ochengi* worms *in vitro*. Auranofin is of particular interest as a macrofilaricidal drug, as it has a 43-fold higher IC_50_ against the microfilariae of *L*. *loa* compared with the IC_50_ for adult female *O*. *ochengi* [26]. This may help overcome a major contraindication for MDA, as severe adverse reactions to the drug-induced death of *L*. *loa* microfilariae is likely in areas where *O*. *volvulus* and *Wuchereria bancrofti* are highly co-endemic with *L*. *loa*.

Our preliminary assays with several anti-helminthic drugs, have already demonstrated that this system, once further validated for reproducibility, could be rolled out as a drug screen for decision making in macrofilaricide drug development programs. Furthermore, the *in vitro* model could support additional studies focused on parasite’s development.

Overall, we are confident that this novel culturing system could offer a promising and much needed platform to study the activity of novel or repurposed drugs against the human filarial parasite *O*. *volvulus*, testing their effects on the growing *O*. *volvulus* worms *in vitro* will support their validation as promising novel and/or repurposed macrofilaricidal drugs.

## Supporting information

S1 Fig Viability (MTT) assay showed dead (A) and live (stained, B) OvL5 worms. The product of MTT reduction (formazan) is blue. Worms were considered dead if no staining or < 50% within the worm was observed using an inverted microscope. Worms stained blue or > 50% stained were considered alive.
(TIF)

S2 Fig Cast (arrow) and live worm after molting of OvL3 to OvL4. (TIF)

S3 Fig Effects of flubendazole and oxfendazole on OvL5 worms. IC50 for inhibition of motility (A) and viability (B) of OvL5s in the presence Flubendazole and IC50 for inhibition of motility (C) and viability (D) of OvL5s in the presence of Oxfendazole.
(TIF)

S4 Fig Effects of niclosamide and nitazoxanide on OvL3 worm. IC50 for inhibition of molting of OvL3s with Niclosamide (A) and Nitazoxanide (B).
(TIF)

S1 Table Information about Ov-biomarkers selected in the study. (XLSX)

S1 Video An example of movements of OvL5 worms—100% motility, constant coiling movement. Motility was recorded every 2–3 days over the full period of the assay.
(MP4)

S2 Video An example of movements of OvL5 worms.- 50% motility, slow and intermittent movement. (MP4)

S3 Video An example of movements of OvL5 worms. 0% motility, no movement. (MP4)
