# Supplementary figures and images for: Development of a preliminary in vitro drug screening assay based on a newly established culturing system for pre-adult fifth-stage Onchocerca volvulus worms
Source: PLoS Negl Trop Dis. 2019 Jan 17;13(1):e0007108. doi: 10.1371/journal.pntd.0007108 (PMC6353222; doi:10.1371/journal.pntd.0007108)

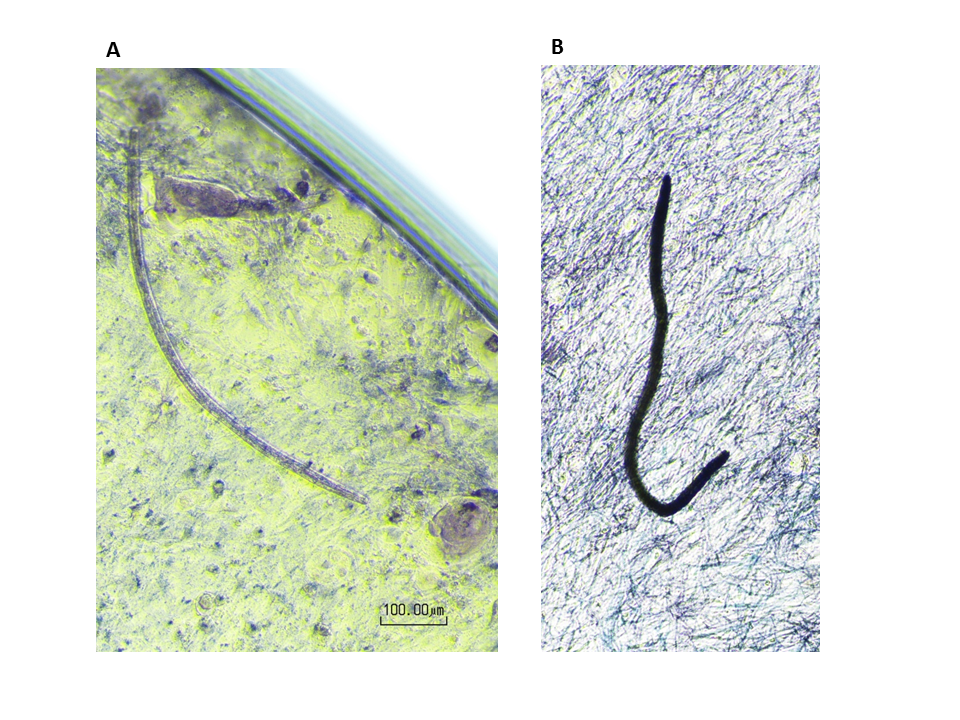

Supplement: S1 Fig — The product of MTT reduction (formazan) is blue. Worms were considered dead if no staining or < 50% within the worm was observed using an inverted microscope. Worms stained blue or > 50% stained were considered alive. (TIF) [file pntd.0007108.s001.tif]

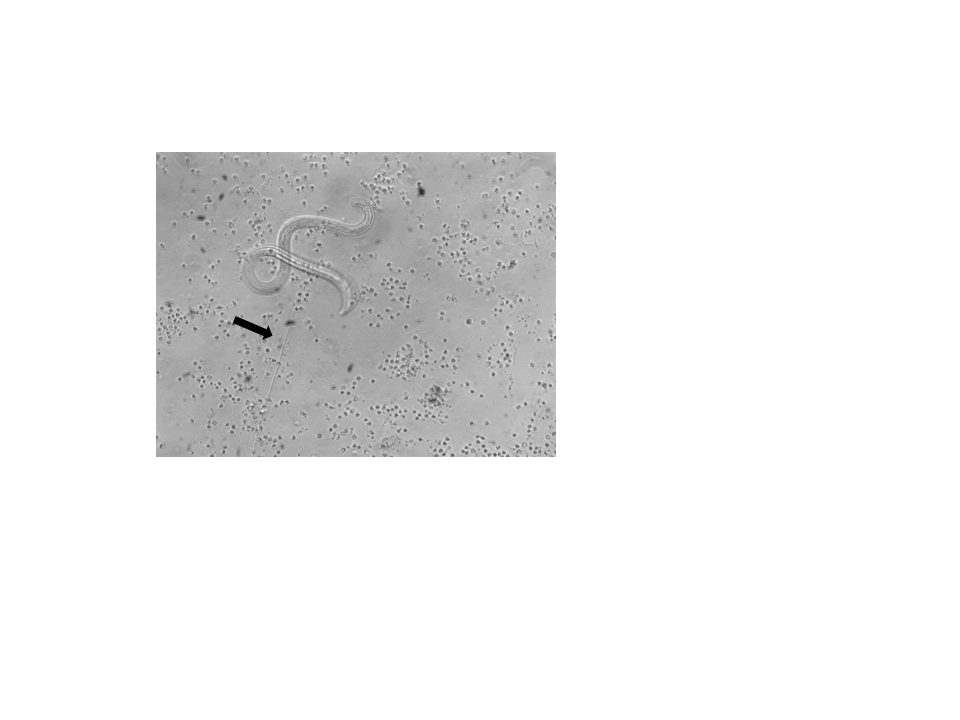

Supplement: S2 Fig — (TIF) [file pntd.0007108.s002.tif]
